# Dramatic increase in consumption of antibiotics in Colombia, 2020-2023

**DOI:** 10.7705/biomedica.7702

**Published:** 2026-03-02

**Authors:** Yeimi López-Mejía, Nelson Alvis, Daniela Paternina, Camilo Guzmán, Francisco Camargo, Manuela Beltrán, Alejandro Sanjuán, Augusto León-Rosso, Salim Mattar

**Affiliations:** 1 Instituto de Investigaciones Biológicas del Trópico, Universidad de Córdoba, Montería, Colombia Universidad de Córdoba Instituto de Investigaciones Biológicas del Trópico Universidad de Córdoba Montería Colombia; 2 Grupo de Investigación en Economía de la Salud, Universidad de Cartagena, Cartagena, Colombia Universidad de Cartagena Grupo de Investigación en Economía de la Salud Universidad de Cartagena Cartagena Colombia; 3 Departamento de Regencia y Farmacia, Universidad de Córdoba, Montería, Colombia Universidad de Córdoba Departamento de Regencia y Farmacia Universidad de Córdoba Montería Colombia; 4 Facultad de Medicina, Universidad del Sinú, Montería, Colombia Universidad del Sinú Facultad de Medicina Universidad del Sinú Montería Colombia; 5 Asesor independiente, Bogotá, D. C., Colombia. Asesor independiente Bogotá D. C Colombia

**Keywords:** Anti-bacterial agents, drug resistance, microbial, health care costs, public health policy, Colombia, antibacterianos, farmacorresistencia microbiana, costos de la atención en salud, políticas públicas de salud, Colombia

## Abstract

**Introduction.:**

Antibiotic consumption and resistance have increased worldwide. Antibiotic resistance results in longer hospital stays and higher healthcare costs.

**Objective.:**

To describe the consumption of antibiotics and associated expenses in Colombia.

**Materials and methods.:**

A meticulous descriptive cross-sectional study of antibiotic consumption and expenditure in Colombia from 2020 to 2023 was conducted. Between 2020 and 2023, a description of the consumption and expenditure of antibiotics in Colombia was made. Data were obtained from IQVIA™ (IMS Health and Quintiles). The prominent families of antimicrobials used in Colombia were selected. Twelve pharmacological families were classified, including 27 antimicrobials and three ß-lactamase inhibitors. The defined daily dose was used to measure antibiotic consumption, identify variations, and evaluate medical prescription practices. The defined daily dose per 1,000 inhabitants per day was estimated to obtain information from the population receiving daily antibiotic treatment. The amount of antibiotics used was estimated in grams and tons per year.

**Results.:**

The top 10 most consumed antimicrobials by defined daily dose per 1,000 inhabitants per day in Colombia were amoxicillin, azithromycin, metronidazole, cephalexin, ciprofloxacin, trimethoprim-sulfamethoxazole, ampicillin, sulbactam, clarithromycin, cefazolin, and dicloxacillin. The total consumption of antibiotics was 2,139 tons, which represented an expense of USD$ 708,112,587, for an increase of 17 and 8%, respectively, during the period.

**Conclusions.:**

The progressive increase in consumption and spending on antimicrobials in Colombia requires a set of interventions that include promoting changes in medical prescribing behaviour and a public education campaign that leads to the adoption of a sustainable public health policy.

## Resumen

**Introducción.:**

Tanto el consumo como la resistencia a los antibióticos han aumentado en el mundo.

**Objetivo.:**

Describir el consumo de antibióticos y el gasto asociado en Colombia.

**Materiales y métodos.:**

Se realizó un estudio descriptivo de cohorte transversal entre el 2020 y el 2023 para analizar el consumo y el gasto de antibióticos en Colombia. Los datos se obtuvieron de IQVIA™ (IMS Health and Quintiles). Se escogieron las principales familias de antimicrobianos más usadas en Colombia. Se clasificaron 12 familias farmacológicas, que incluyeron 27 antimicrobianos y 3 inhibidores de betalactamasas. Se usó la dosis diaria definida para medir el consumo de antibióticos, identificar variaciones, y evaluar prácticas de prescripción médica. Para obtener la información de la población que recibió tratamiento diario con cada antibiótico, se estimó la dosis diaria definida por 1.000 por habitantes por día. Las cantidades usadas de antibióticos se estimaron en gramos y toneladas.

**Resultados.:**

Los diez antimicrobianos más consumidos en Colombia, según la dosis diaria definida por 1.000 habitantes por día, fueron amoxicilina, azitromicina, metronidazol, cefalexina, ciprofloxacina, trimetoprim-sulfametoxazol, ampicilina-sulbactam, claritromicina, cefazolina y dicloxacilina. El consumo total de antibióticos fue de 2.139 toneladas, que representó un gasto de USD$ 708'112.587, para un incremento del 17 y el 8 %, respectivamente, durante el período.

**Conclusión.:**

El incremento progresivo del consumo y del gasto en antimicrobianos en Colombia requiere un conjunto de intervenciones que incluyan promover cambios en el comportamiento de la prescripción médica y una campaña de educación pública que conduzca a la adopción de una política de salud pública sostenible.

According to estimates from the Global Burden of Antimicrobial Resistance study, in 2019, there would have been 4.95 million (95% CI: 3.62-6.57) deaths associated with antimicrobial resistance in the world, of which 1.27 million (95% CI: 0.91-1.71) would be directly attributable to it ^1^. By 2050, infections caused by resistant bacteria could cause 10 million deaths annually ^2^. In contrast, according to the World Bank, the economic impact of antimicrobial resistance in 2050, the annual global gross domestic product would probably be reduced by 1.1%. In the high-impact antimicrobial resistance scenario, the world would lose 3.8% of its gross domestic product by 2050, whereas by 2030, the annual deficit would be $ 3.4 trillion ^3^. Antimicrobial resistance results in extended hospital stays, higher healthcare costs, and even death. This is because more follow-up treatment is required, which generates serious side effects and prolongs patient recovery. Therefore, antimicrobial resistance it is a threat to global public health ^4^.

According to the World Health Organization (WHO), 75% of patients hospitalized with COVID-19 during the pandemic were administered antibiotics as prophylaxis to avoid possible bacterial coinfections ^5^. Patients with severe and critical cases of COVID-19 reported a global average of 81% antibiotic use; however, only 8% of patients suffered from bacterial co-infections which required antibiotic treatment ^5^.

Between 2000 and 2015, antibiotic consumption worldwide increased by 65%, from 21.1 to 34.8 billion defined daily doses. The defined daily dose per 1,000 inhabitants per day increased from 11.3 to 15.5 (39%) ^5^^,^^6^. Countries with high antibiotic consumption have higher antimicrobial resistance levels. Therefore, improving antibiotic use has been an important goal of WHO's global action plan on antimicrobial resistance ^5^^,^^6^.

In Mexico, it has been estimated that pneumonia costs due to antibiotic use represent 16% of the intensive care unit costs. When pneumonia is caused by multidrug-resistant bacteria, the cost of clinical management increases by nine ^7^. In Brazil, the highest direct costs of hospital purchases are antimicrobials compared to drugs for cancer treatment ^8^.

In Colombia, according to the WHO Global Antimicrobial Resistance and Use Surveillance System (GLASS) in 2021, the most consumed antibiotics were beta-lactams/penicillin (33.2%), macrolides/lincosamides/streptogramins (19.9%), and quinolones (10.3%) ^9^. In 2018, the defined daily dose per 1,000 inhabitants per day of the beta-lactams/penicillin group was 6.38, and in 2021, it was 8.78, which shows an increase in consumption ^9^. In addition, the group of macrolides, lincosamides, and streptogramins increased from 3.44 defined daily dose per 1,000 inhabitants per day in 2018, to 5.28 in 2021 ^9^.

Due to the lack of publications regarding the consumption of the main antimicrobials and the associated expenses, this study aimed to describe the consumption of antibiotics in Colombia and the associated expenses.

## Materials and methods

### Study design, data collection, and study population

A meticulous descriptive cross-sectional study of antibiotic consumption and expenditure in Colombia was done for the 2020-2023 period. Data on antibiotic consumption and associated expenditure were obtained from IQVIA™ (IMS Health and Quintiles), a renowned multinational company focused on the use of data, technology, and advanced analytics of pharmaceutical data from commercial and healthcare sources, including drug distributors, pharmacies, insurers, and healthcare providers.

The reference population for all per capita calculations was the general population of Colombia; national population projections provided by the *Departamento Administrativo Nacional de Estadística* (DANE) were used ^10^.

### Selection of antimicrobials

The leading families of antimicrobials most used in Colombia were chosen. The generic names were used to classify them into 12 pharmacological families that grouped 27 antimicrobials and three beta-lactamase inhibitors. The antimicrobials used were aminobenzyl penicillins (amoxicillin, ampicillin), narrow-spectrum penicillins (dicloxacillin), cephalosporins (cephalexin, cefazolin, cefradine, ceftriaxone, cefepime); betalactamic plus betalactamases inhibitors (amoxicillin and clavulanic acid, ampicillin and sulbactam, piperacillin and tazobactam); carbapenems (imipenem, meropenem, ertapenem); aminoglycosides (gentamicin, amikacin); macrolides (erythromycin, clarithromycin, azithromycin); oxazolidinonas (linezolid); fluoroquinolones (ciprofloxacin, norfloxacin, levofloxacin, moxifloxacin); sulphonamide (trimethoprim and sulfamethoxazole); tetracyclines (tetracycline, doxycycline); glycopeptides (vancomycin); phenicoles (chloramphenicol) and imidazole (metronidazole).

### Units of measures of antimicrobial use and expenses

To better understand the use of antimicrobials, the defined daily dose was used. This measure allowed us to determine the consumption of antibiotics, identify their variations, and evaluate medical prescription practices. The defined daily doses compare antimicrobial consumption between regions and countries ^11^. The Anatomical Therapeutic Chemical/Defined Daily Dose (ATC/DDD) methodology approved by the World Health Organization (WHO) was followed ^12^. Consumption was analyzed by calculating the defined daily dose of antibiotics in the study period. The defined daily dose per 1,000 inhabitants per day was calculated to obtain information on the population receiving daily antibiotic treatment. The consumption of in-hospital and out-of-hospital antibiotics was not differentiated, nor was the route of administration. Spending on antimicrobials is given in U.S. dollars. The amount of antibiotics was calculated in grams and tons.

### Data analysis

Antibiotic consumption data were calculated based on defined daily dose and defined daily dose per 1,000 inhabitants per day. The data were analyzed using descriptive statistics tools in Microsoft Excel®. Doses were quantified in grams and tons, respectively.

### Ethical aspects

The present study was conducted with the utmost respect for privacy and confidentiality. Data were anonymized for analysis using code numbers for pharmaceutical, distributing, or marketing companies, names of pharmacies, clinics, hospitals, or health insurance companies, ensuring complete anonymity.

## Results

**Figure 1:**
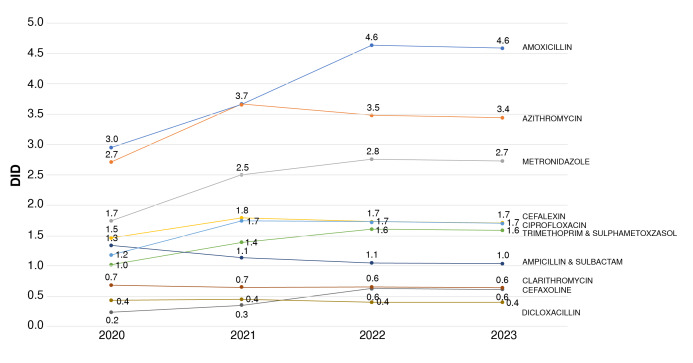
Distribution of daily doses of the ten most used antibiotics

**Figure 2:**
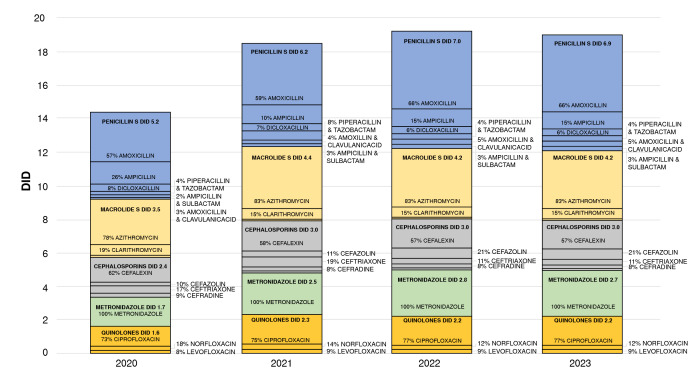
Distribution of daily doses by group

Between 2020 and 2023, the ten most consumed antimicrobials by defined daily dose per 1,000 inhabitants per day in Colombia were amoxicillin, azithromycin, metronidazole, cephalexin, ciprofloxacin, trimethoprim-sulfamethoxazole, ampicillin-sulbactam, clarithromycin, cefazolin and dicloxacillin (figures 1 and 2) (table 1). aminobenzyl penicillins and beta-lactamase inhibitors showed an increase in defined daily dose per 1,000 inhabitants per day (figure 2). In this period, amoxicillin had the highest consumption, which increased from 3.0 to 4.6.

**Table 1:** Antibiotic consumption in Colombia, 2019-2023

Antibiotics	DID				Tons				Millions (USD)			
	Ranking	Total	Stake	Rate of variation	Ranking	Total	Stake	Rate of variation	Ranking	Total	Stake	Rate of variation
			(%)	(%)			(%)				(%)	(%)
Amoxicillin	1	15.9	20	55	1	420	20	34	2	76.1	11	53
Azithromycin	2	13.3	16	27	9	69	3	0	1	94.5	13	-5
Metronidazole	3	9.7	12	57	3	258	12	36	10	24.1	3	19
Cefalexin	4	6.7	8	17	4	233	11	-7	3	58.7	8	12
Ciprofloxacin	5	6.4	8	44	6	96	4	58	8	32.4	5	5
Sulfamethoxazole/ Trimethoprim	6	5.6	7	56	13	38	2	19	12	22.3	3	5
Ampicillin	7	4.6	6	-22	5	191	9	28	14	17.7	3	6
Clarithromycin	8	2.6	3	-6	18	22	1	-34	7	32.4	5	-16
Cefazolin	9	1.8	2	158	8	87	4	51	16	14.7	2	46
Dicloxacillin	10	1.7	2	-7	10	62	3	-13	19	12.9	2	-15
Ceftriaxone	11	1.7	2	-19	11	61	3	-16	21	11.3	2	9
Piperacillin/Tazobactam	12	1.3	2	57	2	299	14	-27	4	56.5	8	-17
Vancomycin	13	1.3	2	90	12	43	2	24	11	22.5	3	49
Norfloxacin	14	1.2	1	-6	19	16	1	-21	24	6.4	1	-19
Amoxicillin/Clavulanic acid	15	1.0	1	114	15	28	1	93	9	32.1	5	105
Cefradine	16	0.9	1	10	14	33	2	-5	20	11.8	2	-2
Gentamicin	17	0.9	1	12	24	4	0	-20	23	8.9	1	-11
Ampicillin/Sulbactam	18	0.8	1	108	7	91	4	139	6	34.1	5	-16
Levofloxacin	19	0.7	1	53	21	7	0	50	18	13.3	2	10
Doxycycline	20	0.6	1	19	20	11	1	-15	17	14.2	2	-5
Meropenem	21	0.4	1	14	17	22	1	6	5	51.3	7	-18
Erythromycin	22	0.4	0	-7	22	6	0	-45	27	2.2	0	-16
Cefepime	23	0.4	0	175	16	23	1	67	22	9.6	1	-21
Amikacin	24	0.2	0	61	25	3	0	13	26	3.4	0	21
Ertapenem	25	0.2	0	160	26	3	0	-13	15	16.6	2	86
Moxifloxacin	26	0.2	0	46	28	1	0	20	25	4.9	1	7
Tetracycline	27	0.1	0	-98	27	2	0	-99	29	1.7	0	-97
Chloramphenicol	28	0.1	0	-6	23	5	0	-14	28	2.1	0	-9
Linezolid	29	0.0	0	3	29	0	0	6	13	18.4	3	37
Cilastatin/Imipenem	30	0.0	0	1	30	0	0	-30	30	0.8	0	-17
**Total**	**30**	**80.7**	**100**	**33**	**30**	**2136**	**100**	**17**	**30**	**708.1**	**100**	**8**

DID: defined daily dose per 1,000 inhabitants per day

Azithromycin defined daily dose per 1,000 inhabitants per day increased from 2.7 to 3.7, but from 2022 onwards, it remained stable between 3.5 and 3.4. Metronidazole was Colombia's third most consumed antibiotic during the study period, with this parameter increasing between 1.7 and 2.7 (figure 1). The daily dose of macrolides increased from 3.5 to 4.2. In 2020, azithromycin had a defined daily dose per 1,000 inhabitants per day of 78%, which increased to 83% by 2023 (figure 2).

Cephalosporins presented an increase in defined daily dose per 1,000 inhabitants per day from 2.4 to 3.0 (figure 2); those with the highest consumption were cephalexin, cefazolin, and ceftriaxone with a variation range of 17, 158, and -19%, respectively (figures 1 and 2) (table 1). A restriction was observed in 2nd, 3rd, and 4th generation cephalosporins for hospital use. In contrast, first-generation cephalexin, administered orally and sold over the counter in pharmacies, is Colombia's fourth most consumed antibiotic (figure 1). Table 1 shows the positive variation in the defined daily dose per 1,000 inhabitants per day of quinolones (ciprofloxacin, levofloxacin, and moxifloxacin); however, norfloxacin showed a negative variation of -6%.

Beta-lactamase inhibitors showed positive variations: amoxicillin and clavulanic acid (114%), ampicillin and sulbactam (108%), and piperacillin and tazobactam (57%). Among the aminoglycosides, amikacin (61%) and gentamicin (12%) showed the most significant variations. Gentamicin (0.9 defined daily dose per 1,000 inhabitants per day) was the most used antibiotic, followed by amikacin (0.2). The tetracycline group was administered: doxycycline at 0.6 and tetracycline at 0.1.

The highest consumption in tons was amoxicillin (420 tons), piperacillin and tazobactam (299 tons), metronidazole (258 tons), cephalexin (233 tons), and ampicillin (191 tons).

**Figure 3:**
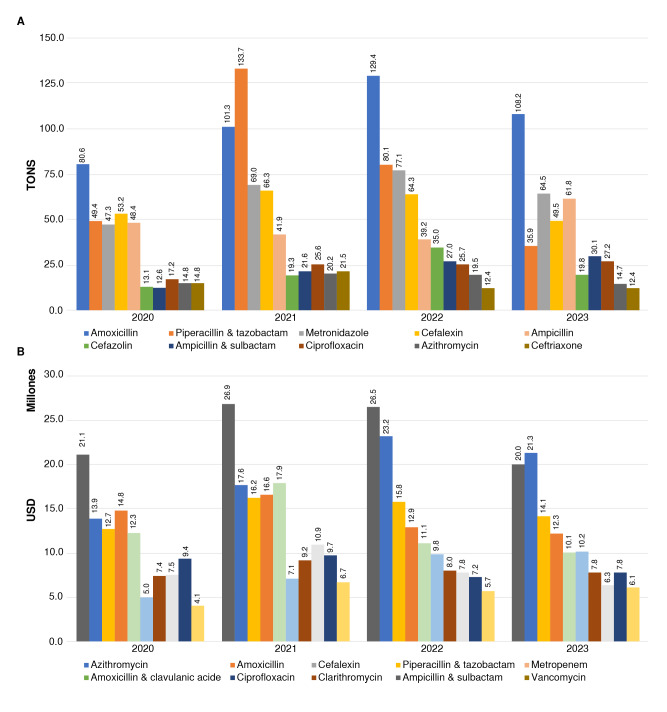
Tons and cost in dollars of the ten most consumed antibiotics

Despite its restrictions in human and animal medicine in Colombia, the consumption of 5 tons of chloramphenicol is striking (figure 3A) (table 1).

Regarding expenditure in dollars, the five antibiotics with the highest expenditure were azithromycin, amoxicillin, piperacillin-tazobactam, cephalexin, meropenem, and ampicillin-sulbactam (figure 3B). Between 2020 and 2023, the associated spending increased to 708,112,587, in Colombia that is an 8% increase (table 1).

In contrast, although carbapenems are exclusively used in hospitals, 25 tons were consumed (meropenem, 22 tons; ertapenem, 3 tons) (figure 3A) . Between 2020 and 2023, Colombia consumed 2,135,930,639 g (2,139 tons) of antimicrobials, representing a 17% increase (table 1).

## Discussion

This study, the first on antimicrobial consumption and its associated expenditure in Colombia, underscores the need for comprehensive interventions. The study revealed a dramatic increase in defined daily doses per 1,000 inhabitants per day and the high expenses it represented for the health system. By 2023, these daily dose related expenses are projected to represent close to 1% of health costs transferred by the Administradora *de los Recursos del Sistema General de Seguridad Social en Salud* (ADRES) for insurers and health service providers in Colombia ^13^. This calls for immediate and comprehensive interventions by all stakeholders.

Figures 1 and 2 depict the global impact of the study, showing the increase in the defined daily dose per 1,000 inhabitants per day of out-of-hospital antimicrobials such as azithromycin, amoxicillin, cotrimoxazole, metronidazole, ciprofloxacin, cephalexin, ampicillin, and dicloxacillin. The surge in azithromycin use during the COVID-19 pandemic was observed worldwide. A systematic review of 19 studies revealed that the average use of antimicrobials in patients with COVID-19 lung infections was 74%, with only 17% of patients having infections. The empirical use of antimicrobial agents is a global concern ^14^^,^^15^.

Similar to our findings in Brazil and Mexico, the highest consumption included penicillins, macrolides, quinolones, and sulfonamides, except for tetracyclines ^16^. The restriction of antimicrobials without a medical prescription was implemented in Brazil and México between 2010 and 2011. However, the impact on the defined daily dose was minimal. México showed better indicators than Brazil for the antimicrobial consumption ^16^. The average consumption of sulfonamides in Brazil did not vary in absolute numbers, with a consumption of 0.8 defined daily doses before and after the regulation change. However, this represented a relative decrease of 2% in total consumption during the study period ^16^. Between 2003 and 2005, in Bogotá (Colombia), a restriction on selling antimicrobials without a medical prescription was implemented. However, its application has not been strict, and the distribution of antibiotics continues without control ^17^.

Regarding the increase in penicillin and cephalosporin consumption, the over-the-counter sale of these antimicrobials and self-medication may have contributed to this increase. Our results were consistent with those of other studies conducted in 2022, in which penicillins were reported as the most used antibiotics in Colombia ^18^. Between 2000 and 2015, the global antimicrobial consumption rate of amino-benzyl penicillins increased by 36% ^9^. This is consistent with GLASS data, which reported penicillin as the most consumed antimicrobial worldwide ^9^. Antibiotic consumption is related to socioeconomic levels, as 56% of antibiotics are consumed in low- and middle-income countries ^19^.

Similarly, another study revealed high rates of dispensing antibiotics without a prescription in the suburbs of Beirut. Consumption rates were higher in low-income socioeconomic areas ^20^. In Europe, antibiotic-resistant bacteria caused 600,000 infections and 27,000 attributable deaths, caused by multidrug-resistant Gram-negative bacteria, secondary to antibiotic used and with huge impact for antibiotic consumption and the prevalence of multidrug resistant infections ^21^. As most antibiotic use occurs in the outpatient setting and a significant fraction is driven by inappropriate use for respiratory infection, the implementation of antimicrobial stewardship programs rationalizes the use of antiobiotics, averts antibiotic misuse/overuse and makes recommendations about antimicrobial resistance threat ^22^. Evidence suggests that countries with high per capita antibiotic consumption have higher rates of antibiotic resistance ^23^^).^ Controlling antibiotic resistance requires reducing antibiotic consumption and addressing socioeconomic factors such as access to drinking water and sanitation, regulation of the private healthcare sector, and a stricter policy on antibiotic control prescription ^24^.

However, few studies have been conducted in Colombia on the use of antibiotics. In a cross-sectional database study, a random sample of patients seen in outpatient clinics was obtained to identify the indications for cephalosporin use in the medical records ^23^. According to clinical practice guidelines, pharmacological variables related to the formulation are considered inappropriate ^25^. Although we did not differentiate out-of-hospital and in-hospital consumption, it is noteworthy that cephalosporins were also one of the most consumed in our study (figures 1-3), cephalexin showed an increase in defined daily dose per 1,000 inhabitants per day consumption of 57%, cefazolin 21 % and ceftriaxone 11%. According to the defined daily dose per 1,000 inhabitants per day, cephalexin's consumption increased by 57%, cefazolins in 21 % and ceftriaxone's in 11% (figure 2) (table 1). Given that cephalexin is taken orally, and there is no restriction on its sale in pharmacies, its use is understandable.

A descriptive study involving intensive care units and other services in Colombian hospitals analyzed the consumption of seven antibiotics (cefepime, ceftriaxone, ciprofloxacin, ertapenem, meropenem, piperacillin, and vancomycin) between 2028 and 2020 ^26^. The results showed an increase in the consumption of ceftriaxone, piperacillin/tazobactam, ertapenem, and cefepime between 2019 and 2020, which is similar to our results in which we analyzed 27 antibiotics and did not discriminate in-hospital use. Furthermore, unlike our study, data from the *Sistema de Vigilancia en Salud Pública* (SIVIGILA) and the *Instituto Nacional de Salud* of Colombia were used, which have under-recording given, that some hospital centers still need to report the consumption of antibiotics ^27^.

Another work in Colombia compared antibiotic prescriptions between capitals cities and municipalities in different regions. Antibiotic prescribing patterns were analyzed by age group, type of antibiotic, and pharmaceutical presentation ^24^.

According to Gaviria-Mendoza *et al.*^25^, 93.5% of the antibiotics prescribed were for oral administration. Penicillins, cephalosporins, and fluoroquinolones were the most commonly used groups of antibiotics whereas amoxicillin was the most frequently prescribed antibiotic; we found a similar result (figures 1 and 2). Their results highlighted significant variations in antibiotic prescribing practices among different demographic groups and regions in Colombia, which may have implications for antibiotic stewardship and public health strategies ^25^. Our work did not analyze regions or forms of presentation.

In 2018, a report from the *Instituto Nacional de Salud* showed increased ciprofloxacin consumption in Colombia's hospitalization services ^27^. Additionally, in 2020, ceftriaxone (9.7 defined daily dose) and ciprofloxacin (9.2 defined daily dose) were the most commonly used antibiotics in services other than the intensive care unit ^28^. These data agree with the results of our study, where ciprofloxacin (6.4 defined daily dose per 1,000 inhabitants per day) was ranked as the fifth most consumed antimicrobial in the country during the study period (table 1).

A comparative study between Costa Rica and Naples (Italy) showed unexpected results. Napoles showed a considerable disparity in gross spending compared to Italy's total spending, while Costa Rica's private sector exhibits even lower gross spending than Italy. The consumption of antibiotics in Italy exceeds that of Costa Rica, with the latter's consumption being 47.70% of Italy's total consumption ^4^. Furthermore, Naples exhibited a 22.43% higher gross expenditure than the Campania, emphasizing the variability in antibiotic use within the same country ^4^. No statistically significant differences were found in antibiotic consumption between regions. Our work did not differentiate the regions in Colombia, which are very different in climate, culture, and population density, and therefore this could be a bias in the present work ^29^.

In Europe and the USA, a study compared the use of antibiotics in outpatients. In Europe and the USA, penicillins, cephalosporins, macrolides, and tetracyclines are the most prescribed in outpatients ^29^. Similar results were obtained in our study regarding penicillins, although they are not wholly comparable since we did not differentiate in-hospital use. The comparison of antimicrobial consumption based on prescription in countries is an essential investigation because it provides information on decision-making in public health policies to control antimicrobials.

Between 2008 and 2012, antibiotic use increased by 22.1% in India. In that country, fluoroquinolones had the highest proportional antibiotic consumption (3.75 defined daily dose per 1,000 inhabitants per day; 24.97%), followed by cephalosporins (13.15%) and macrolides (12.81%) ^20^. In contrast, in our study, consumption of penicillins, macrolides, and cotrimoxazole was higher (figure 2).

Metronidazole is one of the ten most consumed antimicrobials in Colombia. In Sierra Leone (Africa), metronidazole was the most commonly consumed oral antimicrobial ^30^. Its high consumption was related to its use during the management of gastrointestinal infections, and due to its free sale, it is easily available for patients. We do not know whether the consumption of metronidazole in Colombia is related to parasitosis and self-medication by citizens.

On the other hand, the worrying consumption of chloramphenicol is evident in pharmaceutical companies' sales, which increased by 84%, almost one ton per year, for a total of 5.5 tons during the study period (table 1). This antimicrobial is restricted for use in humans and animals by the FDA in the USA due to its adverse effect on the bone marrow. In Colombia, its use in beef cattle has been prohibited since 1981 (ICA resolution 1326) ^31^. However, the drug is freely available for sale in Colombia. The consumption found in our work belongs to a large percentage of the veterinary sector, where no prescription is needed, and it is used in pigs, cattle, and birds. The consumption of animal meats with chloramphenicol is a high risk for human public health, and veterinary doctors must be aware that the use of antibiotics is an essential concept in evidence-based medicine.

Carbapenems and vancomycin were the antibiotics with the lowest consumption rates in this study. This may be due to the fact that their administration occurs exclusively in hospital settings. Since they are administered parenterally, the aminoglycosides gentamicin and amikacin did not increase their consumption. Although this study did not differentiate hospital consumption, it is observed that the highest expenditure falls on orally administered antimicrobials such as azithromycin, amoxicillin, cephalexin, ciprofloxacin, clarithromycin, cotrimoxazole, and metronidazole.

During the COVID-19 pandemic, Arab League countries experienced an increase in the empirical use of antibiotics, such as azithromycin, amoxicillin, and levofloxacin. This has favored the emergence and dissemination of multi-resistant bacteria, such as carbapenem-resistant *Acinetobacter baumannii, Klebsiella pneumoniae,* and *Pseudomonas aeruginosa*^2^. However, in Latin America, *P. aeruginosa* has recorded the highest levels of multi-resistance (41.1%) ^32^. In Colombia, between 2020 and 2021*, K. pneumoniae* was the main pathogen in intensive care units, with resistance rates of 31.2% to third-generation cephalosporins and of 17.2% to carbapenem ^33^. These data reinforce the need to strengthen epidemiological surveillance and strictly regulate the prescription of antibiotics in both human and veterinary medicine.

The limitations of our study are the variable infection rate in consecutive years, with a low (or high), resulting in a variable pattern of antibiotic consumption data, plus the relationship with seasonal impact and different etiologies of infections when inappropriate prescriptions are made. Another point is that public policies vary between regions in Colombia because of campaigns on rational antibiotic use and restriction of free sale, with different results in antibiotic use and resistance in ambulatory and hospitalized patients and the indiscriminate use of animal medicine. Third, the duration of antibiotic use was difficult to monitor in outpatients to document adherence and the number of days they received antibiotic therapy, which limited the reporting of this indicator within the study. We hope that the results obtained in the present study encourage future studies to solve the limitations found in our work.

In conclusion, the study analyzes the spending and consumption of antibiotics in Colombia between 2020 and 2023. Colombia spent USD$ 708 million and consumed 2,136 tons of antibiotics during the studied period. These figures indicate a high level of antimicrobial consumption, which has important implications for antimicrobial resistance and public health. Implementing comprehensive interventions that include changes in medical prescribing, pharmacy involvement, and monitoring systems is essential to ensure compliance and public education campaigns to promote sustainable policy. Our study could provide a first benchmark to measure the impact of interventions aimed at reducing antibiotic use.

We suggest the establishment of rigorous public policies for hospitals, outpatient services, and veterinary settings. These policies include rationalizing drug use, recommendations for administration frequency, optimal dosages based on patient morbidity, and limiting the over-the-counter sale of antibiotics in pharmacies. This should be done in conjunction with scientific recommendations from government agencies.
